# Phase Formation of Iron-Based Superconductors during Mechanical Alloying

**DOI:** 10.3390/ma15238438

**Published:** 2022-11-27

**Authors:** Vladimir A. Vlasenko, Alena Yu. Degtyarenko, Andrei I. Shilov, Alexey Yu. Tsvetkov, Lyudmila F. Kulikova, Alexey S. Medvedev, Kirill S. Pervakov

**Affiliations:** 1 V.L. Ginzburg Centre for High-Temperature Superconductivity and Quantum Materials, P.N. Lebedev Physical Institute of the Russian Academy of Sciences, 53, Leninsky Ave., 119991 Moscow, Russia; 2 L.F. Vereshchagin Institute for High Pressure Physics of the Russian Academy of Sciences, 14, Kaluzhskoe Highway, 108840 Moscow, Russia

**Keywords:** iron-based superconductors, IBS, 122, mechanical alloying, bulk synthesis

## Abstract

We successfully synthesized bulk Ba_0.6_Na_0.4_Fe_2_As_2_ and Sr_0.5_Na_0.5_Fe_2_As_2_ compounds by high-energy mechanical alloying (MA) technique. The MA process results in homogeneous amorphous phases of BaFe_2_As_2_ and SrFe_2_As_2_. It was found that the optimum time for high-energy milling in all cases is about 1.5–2 h, and the maximum amount of amorphous phase could be obtained when energy of 50–100 MJ/kg was absorbed by the powder. After a short-term heat treatment, we obtained nearly optimum sodium-doped Ba_1−x_Na_x_Fe_2_As_2_ and Sr_1−x_Na_x_Fe_2_As_2_ superconducting bulk samples. Therefore, MA is a potential scalable method to produce bulk superconducting material for industrial needs.

## 1. Introduction

In 2008, a novel class of compounds—iron-based superconductors (IBS) with intriguing distinctive properties was discovered [1], and gained interest for the field of practical application. The very first of this class of superconducting compound, LaFeAsO_1-x_F_x_ (1111), exhibits superconductivity at 26 K. However, over several years this class of compounds has expanded considerably to a variety of families, and superconducting critical temperature been raised to 58 K for the SmFeAsO_1−x_F_x_ [2]. The BaFe*_2_*As_2_/SrFe*_2_*As_2_ (122) IBS family superconductors demonstrate critical temperatures (T_c_) up to 37–38 K, high upper critical magnetic field H_c2_ more than 100 T, low anisotropy (γ = 1–2), and high J_c_ (about 10^6^ A/cm^2^) [3,4,5,6], which make them appropriate candidates for practical use [7,8]. The 1111 IBS family is another possible candidate for industrial application. To date, investigations conducted in this family have revealed a very high upper critical field of more than 100 T, and high critical current of more than 10^6^ A/cm^2^ [9]. However, the main disadvantages of this family are the rather complicated synthesis by high-pressure techniques and high anisotropy γ∼5–15 [10,11].

The majority of IBS parent stoichiometric compounds, with the exception of 111 and 1144 systems [12,13], do not show superconducting properties [14]. For instance, the iron-based superconductors of the 122 family, which is one of the most promising materials for practical application due to their outstanding properties [3,15], exhibit superconductivity only with doping: hole-doping when K/Na atoms partially substitute for Ba sites [16,17], electron-doping when Co/Ni atoms substitute for Fe sites [18,19], and isovalent doping when P atoms substitute for As sites [20], or when Fe positions substituted by Ru atoms [21]. Since 122 superconductors are stable at ambient conditions and form easily with any kind of doping, they can be widely used for practical application such as core material for superconducting wires, high-field magnets for particle accelerators, fusion, and other large-scale research facilities. To date, several successful attempts have been made to manufacture long-length superconducting wires [22,23] and pancake coils from IBS [24,25]. The following investigations showed that they have stable transport characteristics and did not degrade up to 30T [26].

Another possible application of IBS concerns thin films production. So far, Ba-122 thin films doped with Co, Ni, and P have been obtained by pulsed laser deposition (PLD) with a sufficiently high critical current [27,28,29]. The high-quality 1111 and hole doped 122 thin films can be produced only by molecular beam epitaxy (MBE) [30,31].

In case of commercial wire and thin film production, the industry should have access to a large amount of high-quality SC material that cannot be produced by laboratory techniques, such as ampoule synthesis, and therefore a scalable method is needed. A mechanical alloying (MA) technique was successfully used to manufacture Ba-122 [32], and 1111 [33] bulk IBS superconductors. The MA method was applied for the preparation of various superconductors in the required amounts: Nb_3_Al [34], MgB_2_ [35], cuprate superconducting compounds [36,37] and others [38,39,40]. In order to develop industrial synthesis technology of the superconducting materials, in this work we use the MA approach for synthesizing hole-doped bulk BaFe_2_As_2_/SrFe_2_As_2_, Ca_0.5_Sm_0.5_FeAsF, and investigate the optimal conditions for the phase formation.

## 2. Methods

Mechanical activation or mechanical alloying is a solid-state powder processing technique for producing high homogeneity ceramic or metallic materials. The MA process kinetics depends mainly on the milling jar volume, ball-to-powder ratio, and initial size of the material [41,42]. The point of MA is in repeated colliding of the material with balls, flattening, cold welding, grinding and rewelding of the particles during high-energy milling. During the process, some amount of powder is trapped and plastically deforms in between the milling balls. As a result, the initial material particles tend to weld together and form aggregates. In case the rate of defects formation exceeds the rate of its relaxation, mechanical activation or mechanical alloying occurs. The following deformation creates conditions for phase formation without continuous heat treatment of the components. However, after the mechanical alloying has been applied, it is often necessary to carry out additional heat treatment of the mixed homogeneous material to provide long-range ordered crystal structure formation. Nevertheless, the heat treatment time after MA is much shorter rather than in the classical solid-state synthesis techniques [41,43]. Therefore, given that MA significantly reduces the subsequent heat treatment time and energy consumption, it seems appropriate to use this method for synthesizing bulk material in large amounts.

In order to produce Ba_1−x_Na_x_Fe_2_As_2_ (BNFA), Sr_1−x_Na_x_Fe_2_As_2_ (SNFA), and Ca_0.5_Sm_0.5_FeAsF (Ca,Sm-1111) bulk samples we used a Fritsch Pulverisette 7 Premium Line (Fritsch GmbH—Milling and Sizing, Idar-Oberstein, Germany) planetary ball mill with tungsten carbide milling garnet. The volume ratio in all cases for milling balls, grinding material and free space was about 1:1:1. The starting reagents were Ba (Lanhit, 99.8%) or Sr (NZRM, 99.97%), Na (Lanhit, 99.99%), CaF_2_ (Reakhim, 99.9%), Sm (NZRM, 99.9%) and precursor FeAs (Fe, Lanhit, 99.99% + As, Lanhit, 99.9999%) synthesized beforehand. For hole-doped Ba_1−x_Na_x_Fe_2_As_2_, Sr_1−x_Na_x_Fe_2_As_2_ we used metallic Ba/Sr, Na and FeAs precursor, and for Ca_0.5_Sm_0.5_FeAsF—metallic Sm, CaF_2_ powder and FeAs precursor, respectively. The bulk compounds were prepared in several steps: reagents were taken in a stoichiometric ratio, placed into the milling jar together with milling balls and closed tightly. After that, the volume of the milling jar was slowly evacuated to avoid pumping out the light powders from the jar down to 10^−3^ Pa by a rotary pump with a LN_2_ trap. The materials in milling jar were placed into the planetary ball mill. The milling process was carried out in several (up to 36) cycles at 800 rpm for 5 min, followed by standing for 3 min for milling garnet cooling. The smaller the size of the milling balls, the longer the cooling time should be. In order to control the MA process, after each 15 min of grinding, the tungsten carbide milling jar was opened inside an argon box, and the mixture was characterized by X-ray diffraction (XRD). The BNFA and SNFA samples with optimum milling time were heat treated for 1 h to restore the long-range ordering of the crystal structure. Considering that the 1111 system is poorly synthesized by the conventional solid-phase reaction method, the synthesis was carried out after MA preparation by a high pressure technique at 50 kbar pressure and 1350 °C in an argon atmosphere [44].

The heat-treated bulk samples were examined by XRD and by magnetic susceptibility measurements. All manipulations were performed in a high purity (>99.998%) argon atmosphere because of the extremely high powder reactivity. The crystal structure and phase composition were studied by XRD with a Rigaku MiniFlex 600 (Osaka, Japan) using Cu–Kα radiation in the 10–90° *2θ* angle region. The XRD data were analyzed using the Rigaku PDXL software (version 2.8.4.0) with PDF-4+ database, and Jana2006 software, using Le Bail and Rietveld method for cell parameters determination [45]. The morphology of milled powders and element analysis was performed using a Scanning Electron Microscope (SEM) JEOL 7001F (JEOL Ltd., Akishima, Tokyo, Japan) with a field-emission cathode and INCA X-Act Energy Dispersive Spectroscopy (EDS) attachment (Oxford Instruments, UK). Superconducting properties of the powder were studied by AC magnetic susceptibility installation (ACMS) and resistivity option in a Quantum Design Physical Property Measurement System (PPMS-9) (San Diego, CA, USA).

## 3. Results and Discussion

**Figure 1:**
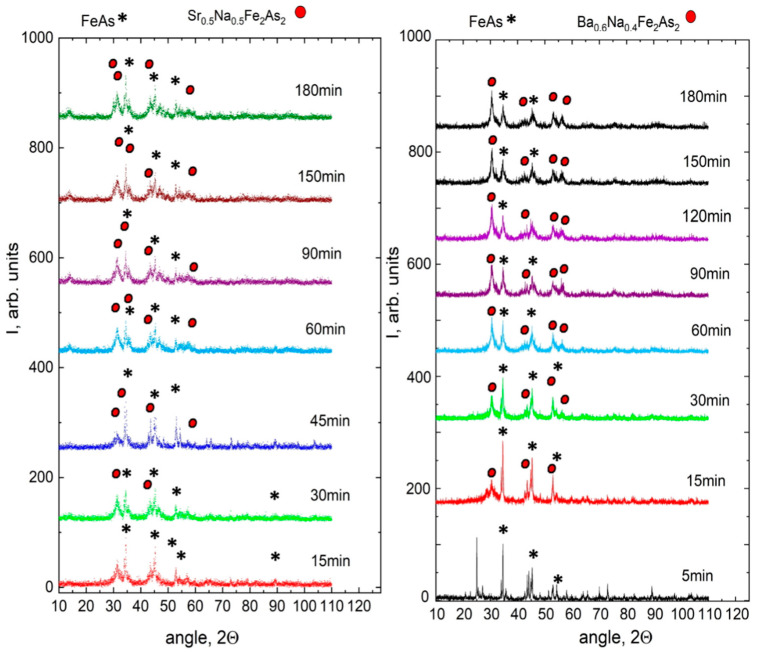
XRD patterns of BNFA and SNFA after various milling times.

By the MA technique, we have successfully produced Ba_1−x_Na_x_Fe_2_As_2_ (BNFA), Sr_1−x_Na_x_Fe_2_As_2_ (SNFA) powders with doping levels of x = 0.4 and 0.5, and Ca_0.5_Sm_0.5_FeAsF powder [44]. XRD measurements show the amorphous 122 phase formation process during the mechanical alloying. However, in the case of the 1111 sample XRD shows rather weak intensity peaks after 150 min of MA. Figure 1 presents XRD patterns for the BNFA and SNFA compounds depending on the milling time. Our data indicate that during MA, the peaks related to the initial materials are suppressed, whereas the peaks associated with the 122 phase appear. Similar behavior for the nickel- and potassium-doped samples were observed previously [43,46].

As mentioned above, during the MA process the hitting tungsten carbide balls mix powder thoroughly, transfer energy to the material, and the initial components start to react. We tried to find the best MA conditions in terms of material energy absorption by milling material per unit mass (*E_BM_*). The *E_BM_* parameter mainly depends on the ball-milling time and milling conditions such as frequency, revolution and rotation radius, ball-to-powder ratio. According to the model presented in Ref. [47], we calculate *E_BM_* using the equation:

(1) $$E_{BM}=c\beta \frac{{\left(\omega_{p}r_{p}\right)}^{3}}{r_{v}}t,$$

where constant c∼0.1, *β* is the mass ratio of balls to the powder, *ω_p_* is the angular frequency, *r_p_* is the revolution radius, *r_v_* is the rotation radius and *t* is the ball-milling time.

**Figure 2:**
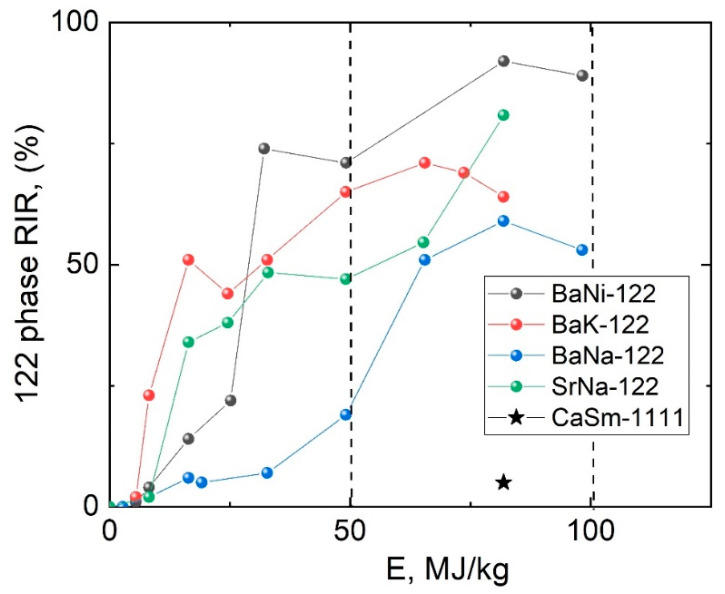
RIR quantitative 122 phase volume analysis for bulk BNFA and SNFA depending on the MA energy.

We estimated the 122 phase volume fractions using Reference Intensity Ratio (RIR); the results are presented in Figure 2. The energy absorption calculated according to the aforesaid formula shows that the greater part of the amorphous Ba-122 phase is formed at *E_BM_* of about 50 MJ/kg (∼1.5 h) and reaches the maximum in the region of 80 MJ/kg (∼2.5 h). It should be noted that according to our experimental data the optimum *E_BM_* for Sr-122 is larger than that for Ba-122 (about 100 MJ/kg). A possible reason of such difference is that the Sr-122 phase formation energy exceeds that for the Ba-122 phase; however further investigation is needed. Further grinding did not show any significant positive effect on the formation of the Ba-122 phase. Similar results were observed for BaFe_2−x_Ni_x_As_2_ superconducting compound [48,49], where the authors claimed that in long term MA the large Ba-122 aggregate appears before the heat treatment, which leads to formation of gaps between the grains, poor grain connectivity of the aggregates and grains [48]. These factors cause deterioration of the overall powder quality and its SC properties.

The Ca_0.5_Sm_0.5_FeAsF powder was treated within 2.5 h (~80 MJ/kg) of MA time. During the MA process, peaks associated with the 1111 phase appear; however, contrary to the 122 phase, the overall 1111 volume appears to be much smaller. It should be noted that we did not obtain superconducting Ca,Sm-1111 phase without preliminary MA treatment.

The element ratio of the Ba_1−x_Na_x_Fe_2_As_2_, and Ca_0.5_Sm_0.5_FeAsF bulk samples obtained from SEM investigations are Ba_0.73_Na_0.27_Fe_2.14_As_1.87_ and Ca_0.56_Sm_0.44_Fe_0.9_As_0.97_F_1.25_, respectively. Data are summarized in Table 1. One can see a deficiency of alkaline metals and arsenic in 122 phases, and arsenic in 1111. A possible reason is the wetting or sticking of grinding balls and jar walls by alkaline metals, and some arsenic sublimation during heat treatment. Taking that into account, an excess of arsenic and alkaline metals should be added. The magnetic susceptibility measurements of the powder after the MA process did not show any superconducting transition in SNFA, BNFA and Ca,Sm-1111, which is similar to BKFA and BFNA samples [43]. After the MA process, we pressed the obtained BNFA and SNFA powders into pellets and heat-treated them for 1 h at a temperature of 850 °C to restore long-range ordering of Ba/Sr-122 phases. High pressure synthesis was used at a pressure of 50 kbar and 1300 °C in an argon atmosphere for Ca_0.5_Sm_0.5_FeAsF. Heat treated samples were examined by SEM and XRD.

**Figure 3:**
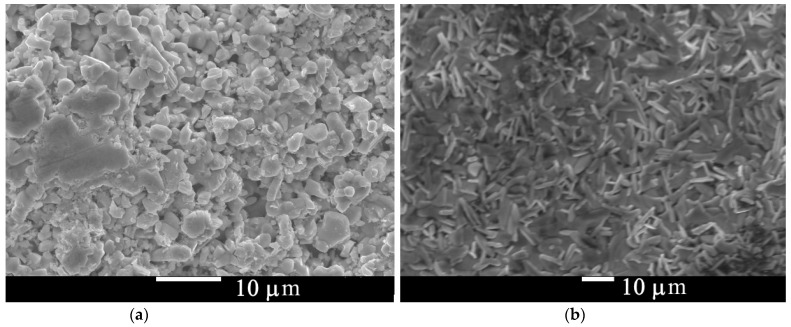
SEM images of the Ba_0.6_Na_0.4_Fe_2_As_2_ (**a**) and Sr_0.5_Na_0.5_Fe_2_As_2_ (**b**) samples heat-treated at 850 °C for 1 h.

**Figure 4:**
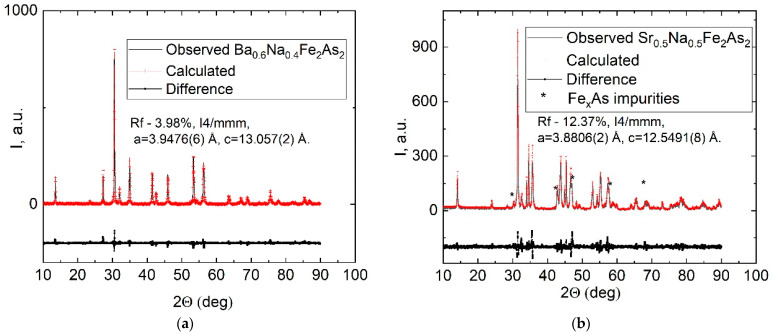
XRD diffraction patterns and Rietveld refinement profile of the Ba_0.6_Na_0.4_Fe_2_As_2_ (**a**) and Le Bail fit of the Sr_0.5_Na_0.5_Fe_2_As_2_ (**b**) powders after annealing.

In Figure 3a,b we present the SEM images of the Ba/Sr-122 pellets surface after heat treatment. In both figures, large number of crystallites with the average size of several µm are observed. Crystallites have different shape—rounded for Ba-122 (Figure 3a) and plate-like for Sr-122 (Figure 3b). This is because of a difference in melting points: Sr-122 has a lower melting point, so it crystallized better at the same temperature. Crystallization indicates the long-range ordering restoration, and high-intensity narrow reflexes should appear in the XRD pattern. The following XRD measurements presented in Figure 4a,b show significant increasing of reflex intensity for BNFA and SNFA after 1 h 850 °C heat treatment. According to our XRD data, the BNFA sample shows almost pure Ba-122 phase, without any visible impurities. In the SNFA sample produced exactly the same way, there is a noticeable amount of Fe_x_As phase impurity.

In the case of Sr_0.5_Na_0.5_Fe_2_As_2_, we found cell parameters to be a = 3.8806(2), c = 12.5491(8) Å with R_p_ = 12.37%, despite the Fe_x_As impurities. The Sr-122 phase volume was about 80%, as was determined from the RIR. In the case of Ba_0.6_Na_0.4_Fe_2_As_2_, we obtained good quality data and refined cell parameters. Composition of the synthesized phase using Rietveld method: a = 3.9476(6), c = 13.057(2) Å (R_f_ = 3.98%). These cell parameters are similar to those obtained in Refs. [50,51]. Moreover, the composition of this phase coincides with EDS data, considering the accuracy of EDS and XRD data.

The diffraction pattern of Ca_0.5_Sm_0.5_FeAsF after heat treatment under high pressure confirms the presence of reflections corresponding to the Ca,Sm-1111 phase [44]. At the same time, the phase content estimated from the diffraction pattern is about 3–5%, which makes it unlikely that there could be proper calculation of its unit cell.

## 4. Superconducting Properties

**Figure 5:**
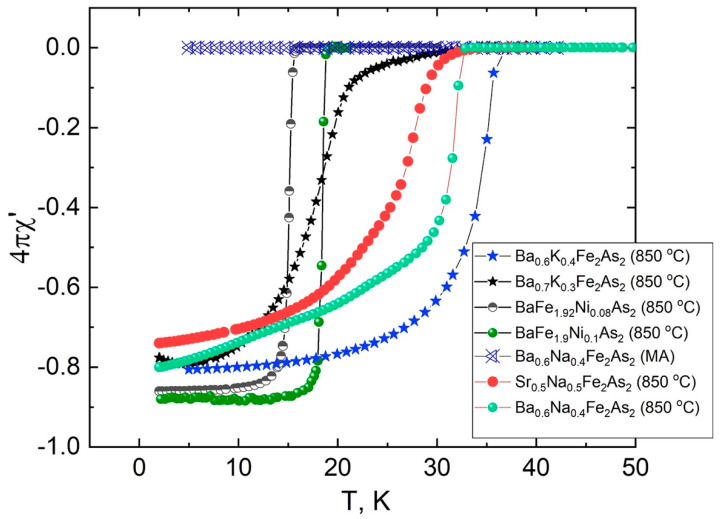
Temperature dependence of the AC susceptibility χ’(T) of Ba_0.6_K_0.4_Fe_2_As_2_, Ba_0.7_K_0.3_Fe_2_As_2_, BaFe_1.92_ Ni_0.08_As_2_, BaFe_1.9_Ni_0.1_As_2_ from our previous work [43] and Ba_0.6_Na_0.4_Fe_2_As_2,_ Sr_0.5_Na_0.5_Fe_2_As_2_ from this work at zero DC magnetic field.

**Figure 6:**
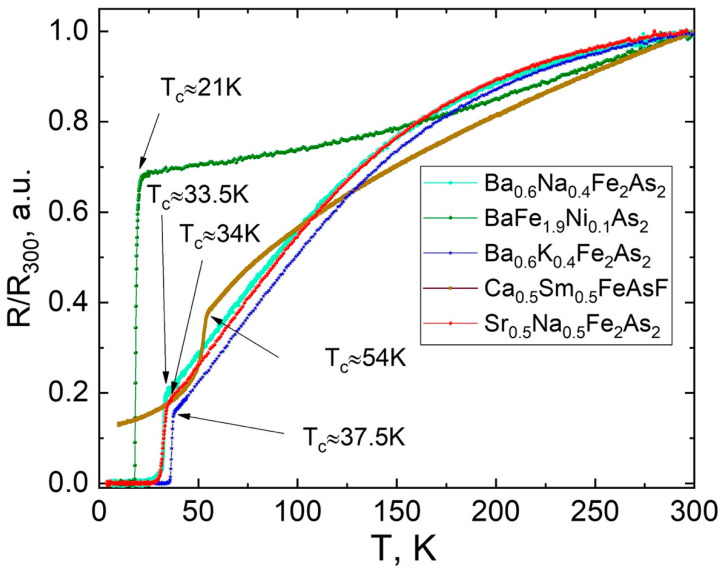
Temperature dependence of the resistivity R (T) of Ba/Sr-122 and Ca,Sm-1111 at zero DC magnetic field.

The superconducting transitions of annealed Ba/Sr-122 samples presented in Figure 5 were studied by AC susceptibility (χ (T), Figure 5) and resistivity (R (T), Figure 6) measurements down to 2 K. In order to avoid form factor of the samples, we normalized R (T) data to resistivity at 300 K. Superconducting transition appears for all heat-treated samples. Superconducting critical temperatures from resistivity measurements correspond to the values of samples of similar composition. The electron doped 122 phase exhibits a very sharp transition and nearly constant susceptibility that follows, which points to a high homogeneity of the sample. The hole-doped Ba/Sr-122 samples show some extension in the low temperature part of the superconducting transition. According to the AC susceptibility measurements, the shielding volume fraction of the electron doped bulks is about 85%, and the hole-doped sample is in the range 70–80% after 1h heat treatment at 850 °C [43]. The values of the critical temperature determined by χ (T) measurements in all 122 samples were slightly lower than in the optimal doped compounds, which was confirmed by the EDS analysis. Therefore, we may conclude that it is necessary to use an excess of the volatile doping material, taking into account the losses during MA and heat treatment.

At the same time, the stoichiometric ratio for electron-doped compounds changes significantly less than for hole-doped ones due to less reactivity and volatility of the components. The Ca_0.5_Sm_0.5_FeAsF sample shows T_c_ ≈ 54 K and a rather low shielding volume fraction about 5% similar to other 1111 compounds. Thus, we show the successful route of synthesizing new 1111 compound under high pressure with preliminary MA treatment [44]. Summarized experimental data are presented in Table 1.

## 5. Conclusions

The effect of material energy absorption on the Ba/Sr-122 phase formation was examined. We show the optimum EBM for amorphous Ba-122 non-superconducting phase formation to be about 50–80 MJ/kg, and for Sr-122 it is about 100 MJ/kg. All electron- and hole-doped samples exhibit superconductivity after short-term annealing. The SEM investigation revealed formation of crystallites of several µm in size, and some arsenic and alkaline metal losses during the MA process and synthesis. Therefore, electron doped samples show sharper superconducting transitions than hole-doped ones. The mechanical alloying method could be a useful technique for fast initial phase formation and large scale, high-quality 122 superconducting powder production.

## Figures and Tables

**Table 1:** Compilation of the Ba/Sr-122 samples EDS, T_c_ and unit cell parameters obtained for different samples.

Chemical Formula	Composition by EDS	T_c_, K		Unit Cell Parameter	
		R(T)	χ’(T)	a, Å	c, Å
BaFe_1.9_Ni_0.1_As_2_ [43]	Ba_1.022_Fe_1.907_Ni_0.093_As_2.003_	21 K	18.5 K	3.9586(2)	12.9820(7)
BaFe_1.92_ Ni_0.08_As_2_ [43] Ba_0.6_K_0.4_Fe_2_As_2_ [43] Ba_0.7_K_0.3_Fe_2_As_2_ [43] Ba_0.6_Na_0.4_Fe_2_As_2_ * Sr_0.5_Na_0.5_Fe_2_As_2_ * Ca_0.5_Sm_0.5_FeAsF [44]	Ba_1.106_Fe_1.921_Ni_0.079_As_1.776_ Ba_0.729_K_0.271_Fe_1.92_As_1.92_ Ba_0.734_K_0.266_Fe_2.15_As_1.99_ Ba_0.73_Na_0.27_Fe_2.14_As_1.87_ -- Ca_0.56_Sm_0.44_Fe_0.9_As_0.97_F_1.25_	- 37.5 K - 33.5 K 34 K 54 K	15 K 36 K 25 K 33 K 33 K 53 K	- 3.9405(7) - 3.9476(6) 3.8806(2) -	- 13.195(3) - 13.057(2) 12.5491(8) -

* This work.

## Data Availability

The data that supports the findings in this study are available from the corresponding author upon reasonable request.
